# Integration of Multifocal Microlens Array on Silicon Microcantilever via Femtosecond-Laser-Assisted Etching Technology

**DOI:** 10.3390/mi13020218

**Published:** 2022-01-29

**Authors:** Bao-Xu Wang, Jia-Xin Zheng, Jin-Yong Qi, Ming-Rui Guo, Bing-Rong Gao, Xue-Qing Liu

**Affiliations:** State Key Laboratory of Integrated Optoelectronics, College of Electronic Science and Engineering, Jilin University, Changchun 130012, China; bxwang_sklio@jlu.edu.cn (B.-X.W.); zhengjx21@mails.jlu.edu.cn (J.-X.Z.); qijy20@mails.jlu.edu.cn (J.-Y.Q.); guomr21@mails.jlu.edu.cn (M.-R.G.); brgao@jlu.edu.cn (B.-R.G.)

**Keywords:** MOEMS, silicon microcantilever, multifocal microlens array, femtosecond laser, dry etching

## Abstract

Micro-opto-electromechanical systems (MOEMSs) are a new class of integrated and miniaturized optical systems that have significant applications in modern optics. However, the integration of micro-optical elements with complex morphologies on existing micro-electromechanical systems is difficult. Herein, we propose a femtosecond-laser-assisted dry etching technology to realize the fabrication of silicon microlenses. The size of the microlens can be controlled by the femtosecond laser pulse energy and the number of pulses. To verify the applicability of this method, multifocal microlens arrays (focal lengths of 7–9 μm) were integrated into a silicon microcantilever using this method. The proposed technology would broaden the application scope of MOEMSs in three-dimensional imaging systems.

## 1. Introduction

Micro-opto-electromechanical systems (MOEMSs) are a new class of micro-systems that integrate micro-optical devices and micro-electromechanical systems (MEMSs); thus, they can simultaneously realize mechanical, electrical, and optical functions [1,2]. With the characteristics of high integration, miniaturizability and accurate control, MOEMSs have great potential applications in optical communication, micro sensing and optical imaging, among others [3,4,5]. Owing to the limitations of fabrication technology, a micro-mirror is the most commonly used micro-optical element in MOEMSs. For example, digital micro-mirror devices are widely used as MOEMS devices in modern optics for digital optical control. To impart more functions in and expand the application scope of MOEMSs, more forms of micro-optical devices need to be integrated with MEMSs, for example, the integration of microlens on MEMS that acts as the optical scanner has wide applications in optical imaging [6,7].

As a basic component in the field of micro-optics, microlenses can realize the properties of focusing, imaging, and beam shaping. Moreover, compared with a single microlens, microlens arrays in integrated systems can obtain information on the multiple positions and angles of images [8,9,10,11]. Therefore, microlens arrays are used in optical applications, including color imaging systems [12], 3D image acquisition systems [13] and fingerprint identification systems [14]. Typically, each microlens in an array has the same size; thus, the focal length of the microlens is the same. Therefore, microlens arrays can only image objects on their common focal plane, resulting in small field-of-view angles and a low depth of field. Based on the properties of microlenses with different focal lengths, the multifocal microlens array can solve the above-mentioned problems, and hence can be applied in 3D imaging systems [15,16,17,18,19]. It is difficult to integrate the above-mentioned micro-optical elements with a complicated morphology and existing MEMSs, e.g., focused ion beam technology can remove materials atom by atom. Thus, high-precision micro/nanostructures can be realized using this technology. Generally, the incident ions only work in the thickness range of several atomic layers on the surface; therefore, the fabrication efficiency of this technology is very low. Photolithography is another common technique used for fabricating micro/nanostructures; however, it has a high requirement for flatness of the sample surface. Therefore, integration of complicated micro-optical elements and MEMS devices is difficult using photolithography. To overcome this, a feasible technology that realizes the integration of micro-optical elements with complicated morphologies and existing MEMSs must be developed.

Femtosecond laser processing [20,21,22] has been widely used to fabricate various types of micro-optical elements because of its ultra-high-precision, mask-free procedure, and in situ processing [23]. However, it has induced high surface roughness in the integration of micro-optical elements and MEMSs of hard materials owing to direct femtosecond laser ablation. In this paper, we proposed a femtosecond-laser-assisted dry etching technology to integrate a multifocal microlens array on the surface of silicon microcantilever, which realizes the integration of micro-optical devices and MEMS devices. A point on the surface of the microcantilever was modified by femtosecond laser, and the modified region was etched and expanded to a microlens via inductively coupled plasma (ICP) etching. Owing to the high degree of freedom of femtosecond laser processing and the effective reduction of surface roughness by dry etching, the fabrication efficiency of microlens arrays with high surface quality at any position on the surface of the silicon microcantilever can be realized. Moreover, a multifocal microlens array was fabricated by adjusting the modification degree of the materials, which can be realized through the adjustment of the laser parameters. Compared with the traditional microlens array, the multifocal microlens array can effectively resolve the problems of capturing 3D image depth of field and numerical aperture in a 3D integral imaging system.

## 2. Materials and Methods

**Figure 1:**
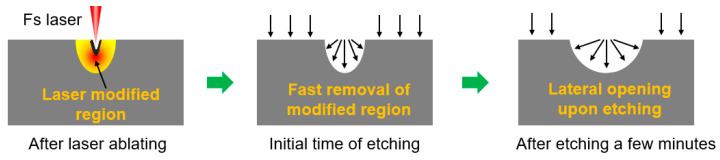
Schematic diagram of fabrication of silicon microlens by femtosecond laser modification with subsequent etching.

**Figure 2:**
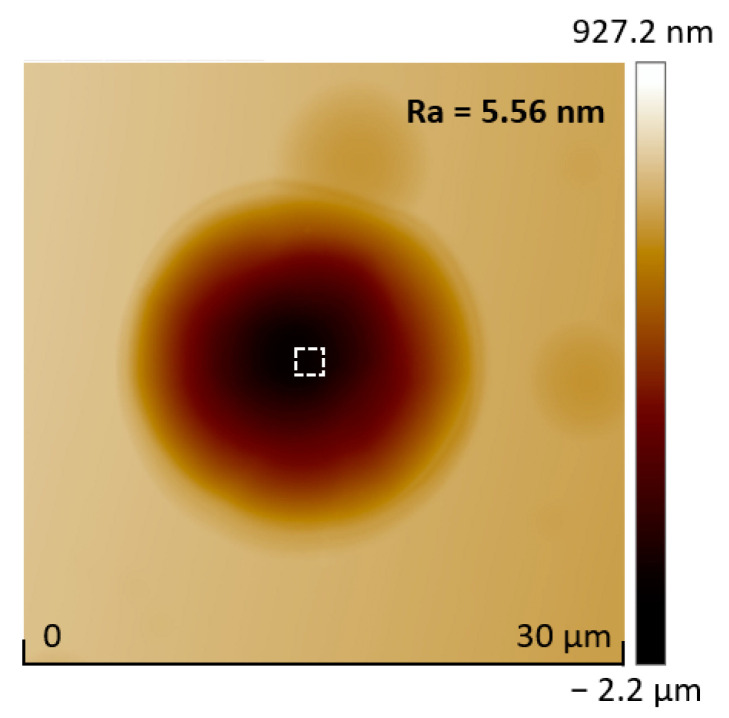
Atomic force microscopy (AFM) image of a silicon microlens fabricated by femtosecond laser modification with subsequent etching.

First, undoped silicon wafers were cleaned with acetone, ethanol, and deionized water for 30 min to obtain a clean surface. The femtosecond laser (343 nm, 200 kHz, 280 fs (Light Conversion Pharos, Vilnius, Lithuania)) is tightly focused through a high numerical aperture objective lens (NA = 0.95, 40×) and matched with a three-dimensional piezoelectric platform (the strokes of the *x*- and *y*-axes are 1.5 mm, that of the *z*-axis is 100 μm, and accuracy is 1 nm) to realize the preparation of micro/nanostructures on the silicon surface [24,25,26,27,28,29]. In addition, the femtosecond laser can also be used to process other materials via multi-photon absorption, for example, lift off GaN [30,31]. After femtosecond laser treatment, a laser-modified region was formed on the silicon surface, changing its physical and chemical properties. Here, the femtosecond laser was used to change the etching rate in a local region with a generation of amorphous and polycrystalline phases, and composition change [23,32]. Then, the silicon sample was etched via ICP (ICP-100A, Tailong Electronics, Beijing, China) with SF_6_ gas at the flow rate of 80 sccm, with the upper electrode power of 500 W, and a lower electrode power of 100 W. In the initial time of the dry etching process, the etching rate of the laser-modified area was greater than that of the unmodified area, and the modified area first etched round holes. With the further progress of etching, owing to the influence of isotropic etching, we can expand outward through the circular hole to obtain a silicon-based microlens with a smooth surface (Figure 1). The surface roughness can be decreased to about 5.56 nm (Figure 2), obtained by measuring the bottom of the microlens.

## 3. Results and Discussions

### 3.1. Preparation of Microlens

**Figure 3:**
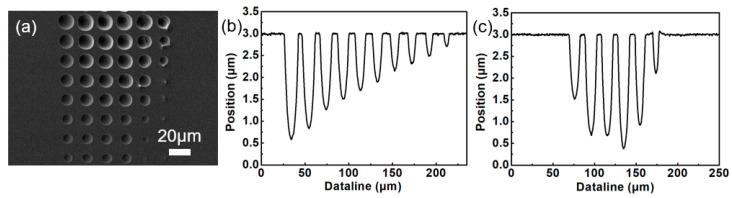
(**a**) Scanning electron microscopy (SEM) images of the multi-focus microlens array; (**b**) Cross-sectional profiles with the depth of lens from (**b**) deep to shallow, and (**c**) shallow to deep and then to shallow.

The dry etching rate of the modified region formed by the femtosecond laser is related to the degree of modification [33,34,35]. By adjusting parameters, such as the power of the femtosecond laser and the number of femtosecond laser pulses, regions with different degrees of modification can be realized. Based on this, we fabricated the microlens arrays with different diameters and depths on silicon wafers. According to the scanning electron microscopy (SEM) result (Figure 3a) and the cross-sectional view of the microlens array (Figure 3b,c), this method can realize the controllable preparation of microlenses with different structural parameters. The actual size of the lens changes periodically according to the experimental expectation, and the lens surface is smooth.

**Figure 4:**
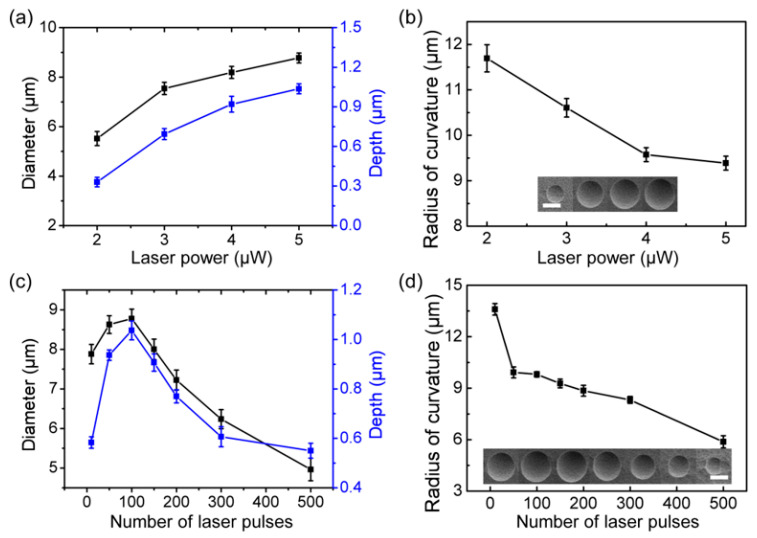
Relationship between the depth and the diameter of structures after etching with (**a**) laser power and (**c**) number of laser pulses. Relationship between the radius of structures after etching with (**b**) laser power and (**d**) number of laser pulses.

From further analysis of the effect of the laser on the etching morphology of the silicon-based microlens, the conclusions are as follows:

(a) Because the modified area increases with the laser power, the size of the silicon-based microlens increases after the ICP etching process. The diameter and depth of the silicon-based microlens showed an increasing trend as the power of the femtosecond laser increased, as shown in Figure 4a. By calculating the diameter and depth of the silicon-based concave microlens under different laser powers, the corresponding radius of curvature was obtained. The relationship between the radius of curvature and laser power is presented in Figure 4b.
(b) With an increase in the laser pulse number, the diameter and depth of the silicon-based concave microlens first increased and then decreased (Figure 4c). This is because the silicon surface would react with oxygen in the air with a greater number of laser pulses, generating a passivation layer on the silicon surface and preventing the etching progress. Therefore, the size of the silicon-based concave microlens decreased with an increase in the number of laser pulses. The different diameters and depths of the silicon-based concave microlens can be obtained by controlling the pulse numbers of the laser. The radius of curvature gradually decreased as the number of pulses increased, as shown in Figure 4d.
(c) The focal lengths of the microlenses with varying radii of curvature also differ. The experimental results show that the size of the silicon-based concave microlens can be flexibly adjusted by changing the femtosecond laser power and pulse number, and the controllable preparation of microlenses with different focal lengths can be realized.

**Figure 5:**
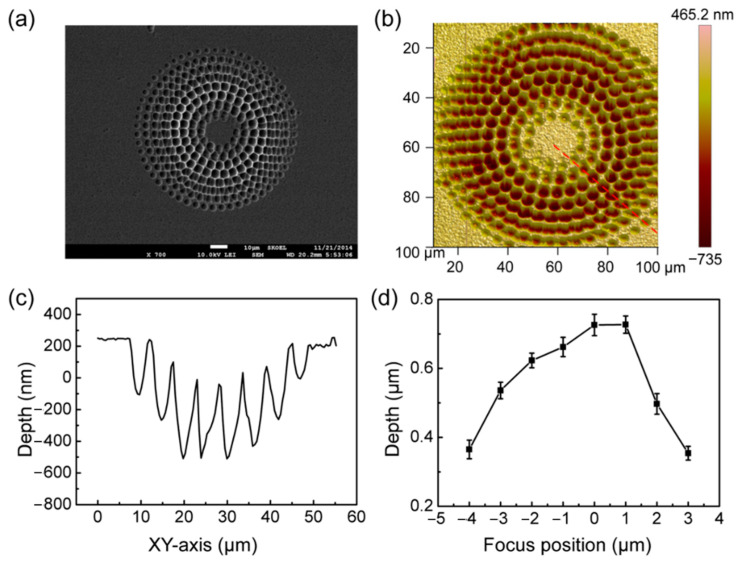
(**a**) SEM and (**b**) AFM images of the concave microlens arrays with different laser focus heights. (**c**) The cross-sectional profile of the concave microlens array. (**d**) Relationship between the depth of the concave microlens array with the laser focus position.

The energy around the laser focus is different and conforms to a gaussian distribution [36,37,38]. Therefore, the degree of the modification region can be affected by the different positions of the focus. The focus position of the silicon surface can be controlled by the movement of the three-dimensional piezoelectric mobile platform, which helps to study the influence of the focus position on the morphology of the microlens after etching. In the experiment, the processing of different laser focus positions was realized by controlling the depth of the silicon sample table. First, the processing was conducted at the laser focus, and gradually deviated from the focus with the processing from inside to outside, and then the circular silicon-based microlens array was obtained via ICP etching. From the SEM image displayed in Figure 5a, the diameter of the final silicon-based concave microlens decreased gradually with the shift in the focus. The atomic force microscope (AFM) image of the silicon-based microlens array in Figure 5b indicates that the depth of the prepared silicon-based concave microlens gradually decreased as the focused energy decreased. A cross-sectional profile of the silicon-based microlens array is shown in Figure 5c. The depth of the silicon-based concave microlens gradually decreased as the focused energy decreased and the processing proceeded outward. The depth of the silicon-based concave microlens was maximum when the silicon surface was at the maximum focus energy, and the depth of the concave microlens gradually decreased with the focus shift (Figure 5d).

**Figure 6:**
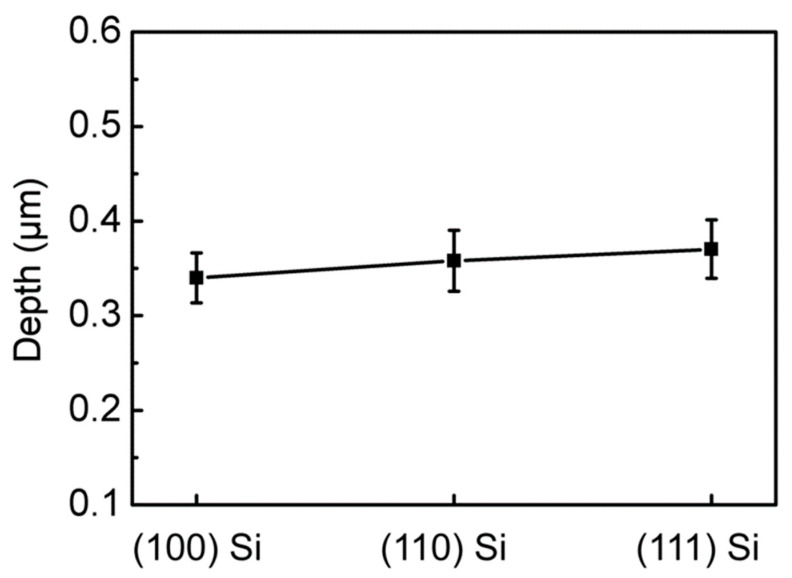
Relationship between the depth of the silicon microlens after etching and three types of silicon with different crystal orientations.

For silicon with different crystal orientations, anisotropy is often observed due to different crystal orientations in the process of wet etching. This leads to an irregular shape when etching a circular structure. To verify the effect of crystal orientation on the etching effect, we replaced silicon wafers with different crystal orientations for the experiments (Figure 6). It was found that the depths of the silicon-based concave microlenses prepared using silicon with three crystal orientations are basically the same, i.e., the morphology of the microlens is not significantly dependent on the silicon crystal orientation. This is because the principle of dry etching involves using the plasma of chemical gas to produce a chemical reaction, and accelerate physical etching and chemical etching through an electric field, so the etching is isotropic. It should be mentioned that by changing the etching gas in the cycle during the etching process, high-aspect-ratio silicon microstructures can be realized [39,40]. Therefore, different patterned silicon-based microlens arrays can be prepared using this method.

### 3.2. Microlens Array Fabrication

**Figure 7:**
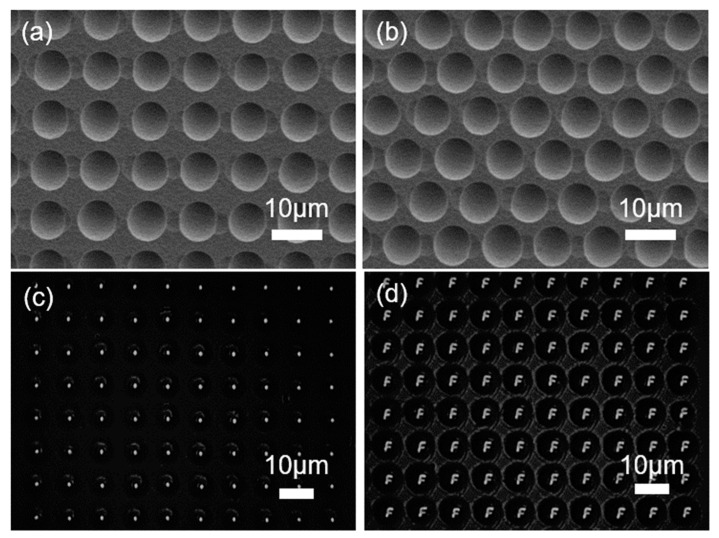
SEM images of (**a**) rectangular microlens array and (**b**) hexagonal microlens array. (**c**) Focal spots and (**d**) optical performance of the hexagonal microlens array.

Femtosecond laser processing has a high degree of freedom, which can plan the trajectory of light spots and then realize the preparation of arbitrary patterned structures. In the experiment, two types of silicon-based microlens arrays were prepared by controlling the movement of a three-dimensional platform. First, the silicon wafer was ablated by a femtosecond laser to form a rectangular arrangement and a honeycomb dense arrangement lattice. Then, the silicon wafer processed using the femtosecond laser was etched via ICP. Owing to the use of the same laser parameters, the modification degree of each part was the same, thus two groups of microlens arrays with the same size and good morphology were formed after dry etching. The two silicon-based microlens arrays were characterized via SEM; the SEM images are shown in Figure 7a,b. Finally, the optical properties of the rectangular array microlens were characterized, as shown in Figure 7c,d. Thus, the silicon-based lens array has good focusing and imaging effects.

**Figure 8:**
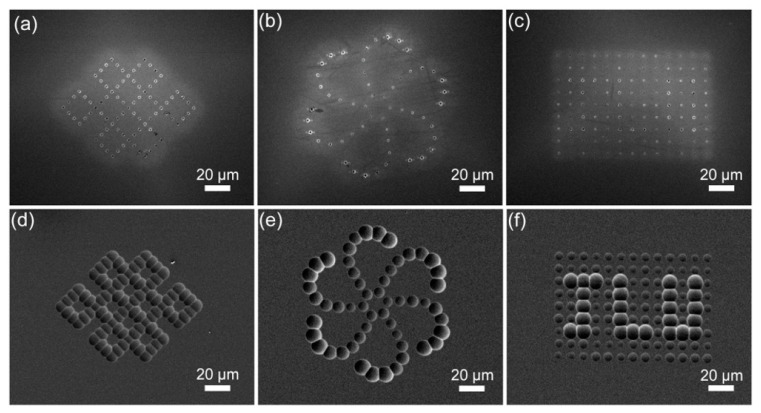
SEM images of (**a**) Chinese knot, (**b**) petal, and (**c**) letters “JLU” before etching; and (**d**) Chinese knot, (**e**) petal, and (**f**) letters “JLU” after etching.

In addition to the above regular microlens array, an arbitrary arrangement of microlenses with inconsistent structures and sizes can be realized by adjusting the laser parameters and scanning path in the experiment. Through this method, some other specific microlenses were fabricated, such as Chinese knots, petals, and hidden letters, as displayed in Figure 8a–c. The corresponding SEM images obtained after etching each pattern are shown in Figure 8d–f. It is difficult to distinguish the final designed microlens array from the laser-modified pattern. After etching, microlenses of different sizes were formed to realize a clear pattern. In particular, the letters “JLU” were difficult to observe after laser modification. Following etching, the hidden letters can be clearly seen by the distinction of the microlens diameter.

### 3.3. Multifocal Microlens Array Integration on Silicon Cantilever

**Figure 9:**
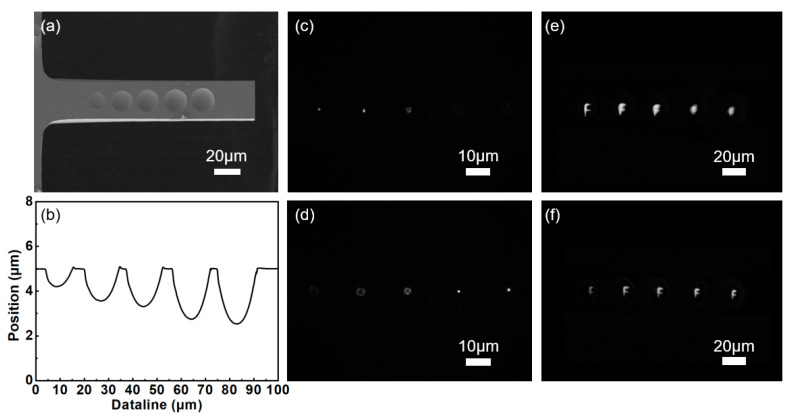
(**a**) SEM image of the silicon-cantilever-integrated multi-focus microlens array. (**b**) The cross-sectional profile of the multi-focus microlens array. (**c**,**d**) Focal spots at different focal locations of the multi-focus microlens array. (**e**,**f**) Optical performance at different focal locations of the multi-focus microlens array.

Femtosecond lasers have the characteristics of local in situ processing and micro nanostructure preparation at any position of a nonplanar structure. Therefore, we can use this method to realize the controllable preparation of a microlens array on a silicon microcantilever. By adjusting the position of the light spot and the degree of modification, we fabricated microlens arrays of different sizes on a silicon microcantilever (Figure 9a). The size of the lens determines its focal length, and its basic relationship satisfies the following equation:

(1) $$R=\frac{h^{2}+r^{2}}{2h}$$

(2) $$f=-\frac{R}{2}$$

where *r* and *h* are the radius and depth of the microlens, respectively; *R* is the radius of curvature of the microlens, and *f* is the focal length of the microlens. According to Equations (1) and (2), the focal lengths of the leftmost and rightmost microlenses were 7 and 9 μm, respectively. Figure 9b presents the cross-sectional characterization curve of the multifocal microlens array. Because of the different focal lengths of the microlenses, the focus morphology and imaging effect also differ at different positions. The focusing and imaging of the lens arrays at different focus positions are depicted in Figure 9c–f. When the large lens is at the focus, the focus and imaging of the rear small lens are unclear. Contrarily, when the small lens is in focus, the large lens will be out of focus. A multifocal microlens array was successfully fabricated on a silicon microcantilever, enabling the integration of micro-optical devices and MEMSs.

## 4. Conclusions

In summary, we investigated the effects of femtosecond laser power and pulse number on the structure of a microlens after etching. It was found that the curvature of the silicon-based concave microlens decreased with an increase in the laser power. With an increase in the number of laser pulses, the diameter and depth of the silicon-based concave microlens first increased and then decreased. The effects of focus change and silicon crystal orientation on the final effect of the experiment were studied. It was found that when the silicon surface is at the maximum focus energy, the depth of the silicon-based concave microlens is at its maximum. With the gradual shift in focus, the depth of the concave microlens gradually decreases. The degree of modification of the laser is unrelated to the crystal direction of silicon. Uniform lens arrays with different arrangements and microlens arrays with arbitrary shapes and sizes were established on the silicon surface. Finally, a multifocal microlens array was fabricated on a silicon microcantilever. Meanwhile, the focusing and imaging effects of the 7 and 9 μm microlenses at different positions were compared. The integration of micro-optical components and MEMSs was realized, providing potential applications for silicon-based MEMSs in the fields of optical communications, digital image processing and biomedicine.

## Data Availability

Not applicable.
